# Flourishing deep-sea AAP bacteria detected by flow cytometric sorting and molecular analysis

**DOI:** 10.1371/journal.pone.0218753

**Published:** 2019-06-19

**Authors:** Dajun Qiu, Liangmin Huang, Xin Liu, Senjie Lin

**Affiliations:** 1 CAS Key Laboratory of Tropical Marine Bio-resources and Ecology, South China Sea Institute of Oceanology, Chinese Academy of Sciences, Guangzhou, China; 2 Guangdong Provincial Key Laboratory of Applied Marine Biology, South China Sea Institute of Oceanology, Chinese Academy of Sciences, Guangzhou, China; 3 University of Chinese Academy of Sciences, Beijing, China; 4 Department of Marine Sciences, University of Connecticut, Groton, Connecticut, United States of America; CAS, CHINA

## Abstract

Pigmented bacteria cells, including aerobic anoxygenic phototrophic (AAP) bacteria, contribute significantly to secondary production and aquatic carbon cycling but their distribution in the deep sea is still not well understood, especially in the South China Sea. In this study, microscopic, flow cytometric, and molecular analyses were carried out to investigate the abundance and diversity of AAP bacteria at seven stations in the South China Sea. The results revealed the existence of bacteriochlorophyll-containing bacteria below 500 m from two of seven stations. Flow cytometric analysis detected red and infra-red fluorescence under blue (488 nm) light excitation from fluorescent cells. Blue light-excited red fluorescence of these cells from the 1000 m depth at station E403 were verified using epifluorescence microscopy. Based on fluorescence and side scatter features, fluorescent cells were sorted and subjected to molecular analysis. DNA was extracted from these sorted cells from both stations for PCR amplification using 16S rDNA primers. Sequencing of the PCR products showed that the sorted cells from the 1000 m depth at station E403 belonged to the genus *Porphyrobacter*. The cell population sorted from 500 m at station E703 contained *Sphingomonas* and a *Methylobacterium-*like taxon. All these three taxa belong to aerobic anoxygenic phototrophic alpha-proteobacteria. Using flow cytometric analysis, we found that the abundance of *Porphyrobacter* sp. at 1000 m was 2.71–2.95×10^4^ cells mL^-1^ whereas cell counts of *Sphingomonas* sp. and *Methylobacterium* at 500 m were about 3.75–4.12×10^5^ cells mL^-1^. These results indicate that albeit not ubiquitous in deep water, bacteriochlorophyll-containing bacteria can be abundant in the deep-sea aphotic zone.

## Introduction

The studies of pigment-containing, noncyanobacterial, phototrophic, bacteria have been increasing in the last decades [1–12]. Of particular interest are aerobic anoxygenic phototrophic (AAP) bacteria, which have become commonly known in both marine and limnic environments and usually show orange, red, pink, brown, or yellow colors due to the presence of various carotenoids [8]. These organisms are usually in low abundance [7,13], but on average their cell sizes are two times larger than regular heterotrophic bacteria in oligotrophic waters [14]. AAP bacteria are capable of utilizing light and organic matter to acquire energy [1]. Some of these bacteria can degrade pollutants [15]. Kolber et al. (2001) found a wide distribution of bacteriochlorophyll-containing, nonphotosynthetic bacteria in surface water, which acquire energy through the oxidation of organic matter [3]. In addition to the light-absorbing bacteria that live in the euphotic zone, pigmented bacteria have also been identified in hydrothermal vents, where surface irradiance cannot reach and these bacteria live photoheterotrophically on low amounts of thermal radiation in the environment [4]. Huber et al. (2007) also found Rhodobacterales and Spingomonadales bacteria in a 1520 m deep sea at an active volcano [16], lineages known to contain bacteriochlorophyll [17,18]. However, to verify that AAP bacteria can exist in high abundance in the relatively cold dark provinces of the deep ocean, it is imperative to investigate whether these bacteria can be detected in high abundance below the euphotic zone. In this study, we used microscopic, flow cytometric, as well as molecular analyses on microbial communities in the deep waters of the South China Sea. We detected a variety of bacteria that contained bacteriochlorophyll at the depths of 500 and 1000 m.

## Materials and methods

### Ethics statement

For any locations/activities for which specific permission was not required, we state that a. no specific permissions were required for these locations/activities; b. that the field studies did not involve endangered or protected species.

### Research cruise and sample collection

**Fig 1:**
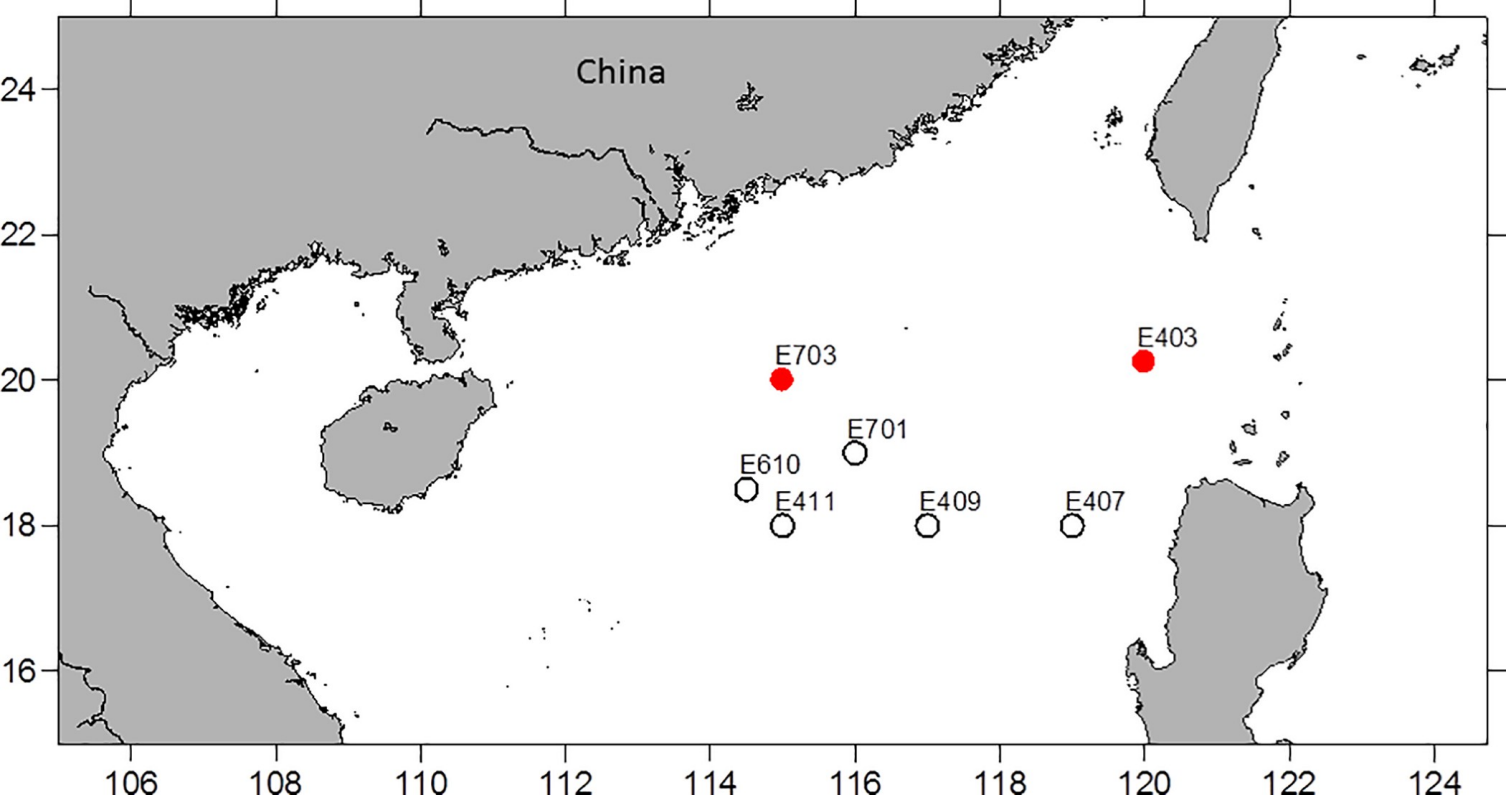
Map of study site. Samples were collected from seven stations, but AAP bacteria were detected only from stations E403 (1000 m) and E703 (500 m).

We sampled deep-sea water from seven stations in the South China Sea (Fig 1, Table 1) onboard research vessel Shiyan III during the South China Sea Institution of Oceanology Open-Cruises in September 2005. Water samples were collected using Niskin bottles attached on CTD rosettes. Four 2 mL subsamples were preserved in 1% formaldehyde, and the sample tubes were immediately covered with tinfoil to avoid fluorescence quenching, kept in room temperature for 10 minutes, and frozen in liquid nitrogen. The samples were stored in liquid nitrogen while onboard and at -80°C as soon as they arrived at our laboratory until analysis (within two months).

**Table 1:** Location, sampling dates and depth for South China Sea stations sampled for deep-sea AAP bacteria studies in 2005.

Station	Longitude	Latitude	Date	Depth- Bottom(m)	Sampling Depth (m)
**E403**	**119˚59.239΄E**	**20˚29.634΄N**	**Sep. 9, 2005**	**1864**	**500, 800, 1000, 1500**
**E407**	**119˚00.222΄E**	**18˚00.035΄N**	**Sep. 10, 2005**	**4041**	**500, 800, 1000, 1500, 2000, 4000**
**E409**	**117˚00.225΄E**	**17˚59.596΄N**	**Sep. 11, 2005**	**3904**	**500, 800, 1000, 1500, 2000, 3000, 3900**
**E411**	**114˚59.661΄E**	**18˚00.062΄N**	**Sep. 12, 2005**	**3569**	**500, 800, 1000, 1500, 2000, 3000, 3500**
**E610**	**114˚29.924΄E**	**18˚29.760΄N**	**Sep. 20, 2005**	**1570**	**500, 800, 1000, 1500**
**E701**	**116˚00.493΄E**	**18˚59.721΄N**	**Sep. 21, 2005**	**1552**	**500, 800, 1000, 1500**
**E703**	**115˚06.161΄E**	**19˚53.968΄N**	**Sep. 22, 2005**	**1052**	**500, 700, 900**

### Flow cytometric analysis

In the laboratory, two of the four subsamples were mixed with 1μm Yellow-Green fluorescence polystyrene beads (Polysciences Inc, Warrington, USA) as the internal standard and subjected to flow cytometric analysis. One of the two samples was analyzed on FACS Calibur (Becton Dickinson, Sparks, USA) to detect red fluorescence (610±20 nm using 488 nm as the excitation wavelength), and to characterize forward scatter and side scatter. The other sample was re-analyzed on a FACSAria (Becton Dickinson, Sparks, USA) to detect infra fluorescence (780+60 nm) under 488 nm excitation and cells were sorted based on infra-red fluorescence and side scatter features. During cell analysis and sorting, an appropriate threshold of side scatter signal was set to separate signals of the fluorescent cells from background noise.

### Microscopic analysis

The fourth subsample from the 1000 m depth was observed under a Leica DMR epifluorescence microscope for morphology and fluorescence. Red fluorescence (> 515 nm) was elicited under blue light excitation (450–490 nm).

### Molecular analysis

The sorted cell samples were centrifuged at 10,000 × g for 10 min and the supernatant was carefully removed. The cell pellet was resuspended in 0.5 mL DNA buffer (10mM Tris pH 8.0; 100 mM EDTA pH 8.0; 0.5% SDS; 200 μg mL^-1^ proteinase K), and incubated overnight at 55°C. Overnight incubation combined with rigorous vortex at the end was done to ensure sufficient lysis of the preserved cells. DNA was isolated using CTAB method and purified using Zymo Clean & Concentrate columns [19]. Bacterial 16S rDNA was amplified using the resulting DNA as the template and primers that were designed based on conserved sequence regions across different bacterial lineages. These primers were bac1 (F): 5' -AGA GTT TGA TCM TGG CTC AG—3' (M = A or C) and bac2 (R): 5' -ACC TTG TTA CGA CTT CAC—3', respectively corresponding to *E*. *coli* 16S rDNA positions 8–27 and 1489–1506 [20]. They were very similar to what has recently been demonstrated to be the most effective universal primer set for bacteria. In parallel, separate PCR reactions were run with universal 18S rDNA primers, and the lack of PCR products was used as indication that the sorted cells do not belong to eukaryotic algae [19]. The 16S rDNA PCR products were purified using Zymo DNA Concentrator and each was cloned into a TA vector. After transformation, resultant clones were randomly picked, their plasmids prepared, and each plasmid was sequenced from both sides on an ABI Prizm 3700 system (Perkin Elmer, Branchburg, NJ, USA). Sequences were trimmed and assembled into a contiguous sequence and BLAST- searched against GenBank databases. Reported sequences hit by our sequences were compiled along with some other sequences from related bacteria for the phylogenetic analysis.

In the phylogenetic analysis, compiled sequences were aligned using CLUSTAL W (1.8) [21]. Neighbor Joining and Maximum Parsimony analyses were performed using the Phylip phylognetic analysis package [22] with 1000 and 500 bootstrap resampling, respectively. For Maximum Likelihood analysis, the datasets were run through ModelTest v3.7 [23] to identify the best-fit nucleotide substitution model. The best-fit model (GTR+G+I) was then used to infer a Maximum Likelihood tree using the PhyML package [24], with nucleotide substitution rates (AC, 0.83378; AG, 1.59680; AT, 1.06771; CG, 0.89531; CT, 3.15111; GT, 1.0 (fixed)), gamma shape parameter (0.264) and proportion of invariable positions (0) optimized based on the data. The reliability of the tree topology was evaluated using the bootstrap support values.

## Results

### Flow cytometric analysis

**Fig 2:**
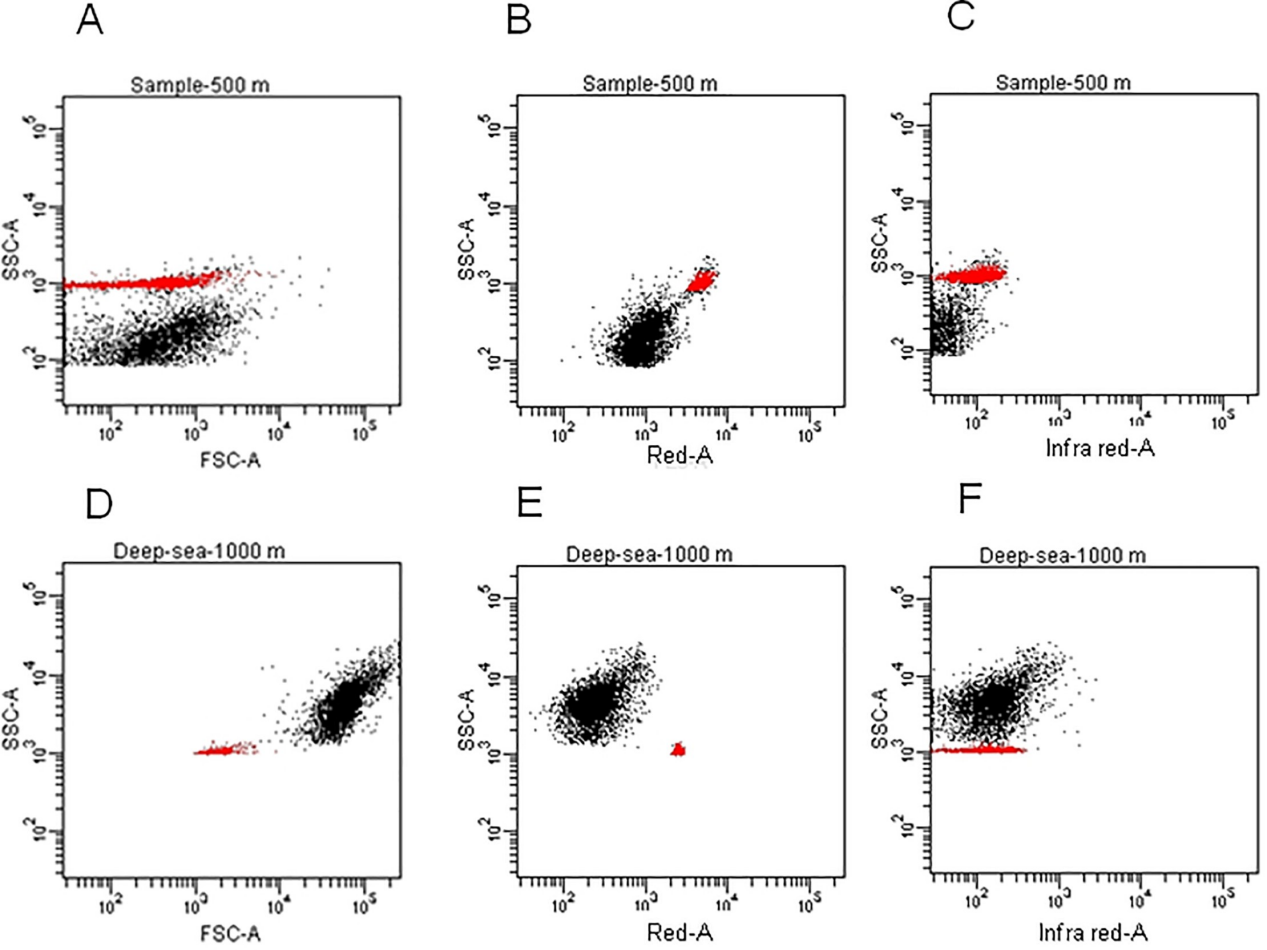
**Plots of flow cytometric analysis results for samples collected at 500 m (A-C) at station E703 and 1000 m (D-F) at station E403.** A, D) side scatter (SSC) versus forward scatter (FSC). B, E) samples side scatter (SSC) versus red fluorescence (610/20 nm). C, F) SSC versus Infra-red fluorescence (780/60 nm). Infrared fluorescence is characteristic of bacteriochlorophyll and cyanobacteria. Red symbols in each plot denote 1μm YG fluorescence beads (Ploysciences, Inc, USA).

From the samples collected at the seven stations onboard R/V Shiyan ш during the South China Sea Institution of Oceanography Open-Cruises in September 2005 (Fig 1), microbial populations emitting red fluorescence were detected from two stations. One population was from the depth of 500 m at station E703 (19˚53.968΄N, 115˚06.161΄E), which emitted strong red fluorescence at 610/20 nm under 488 nm excitation (Fig 2A). These pigmented cells were re-analyzed under the FACSAria cytometer and were found to emit dim infra-red fluorescence at 780/60 nm (Fig 2B), a characteristics of bacteriochlorophyll [25] and cyanobacteria [26]. The cell concentration of this population was about 3.75–4.12×10^5^ cells mL^-1^. Another cell population, which also emitted fluorescence in the red (Fig 2C) and infra-red spectra (Fig 2D), was identified from station E403 (20˚29 .634΄N, 119˚ 59.239 ΄E) at 1000 m deep. The abundance of this cell population was approximately 2.71–2.95×10^4^ cells mL^-1^.

### Microscopic analysis

**Fig 3:**
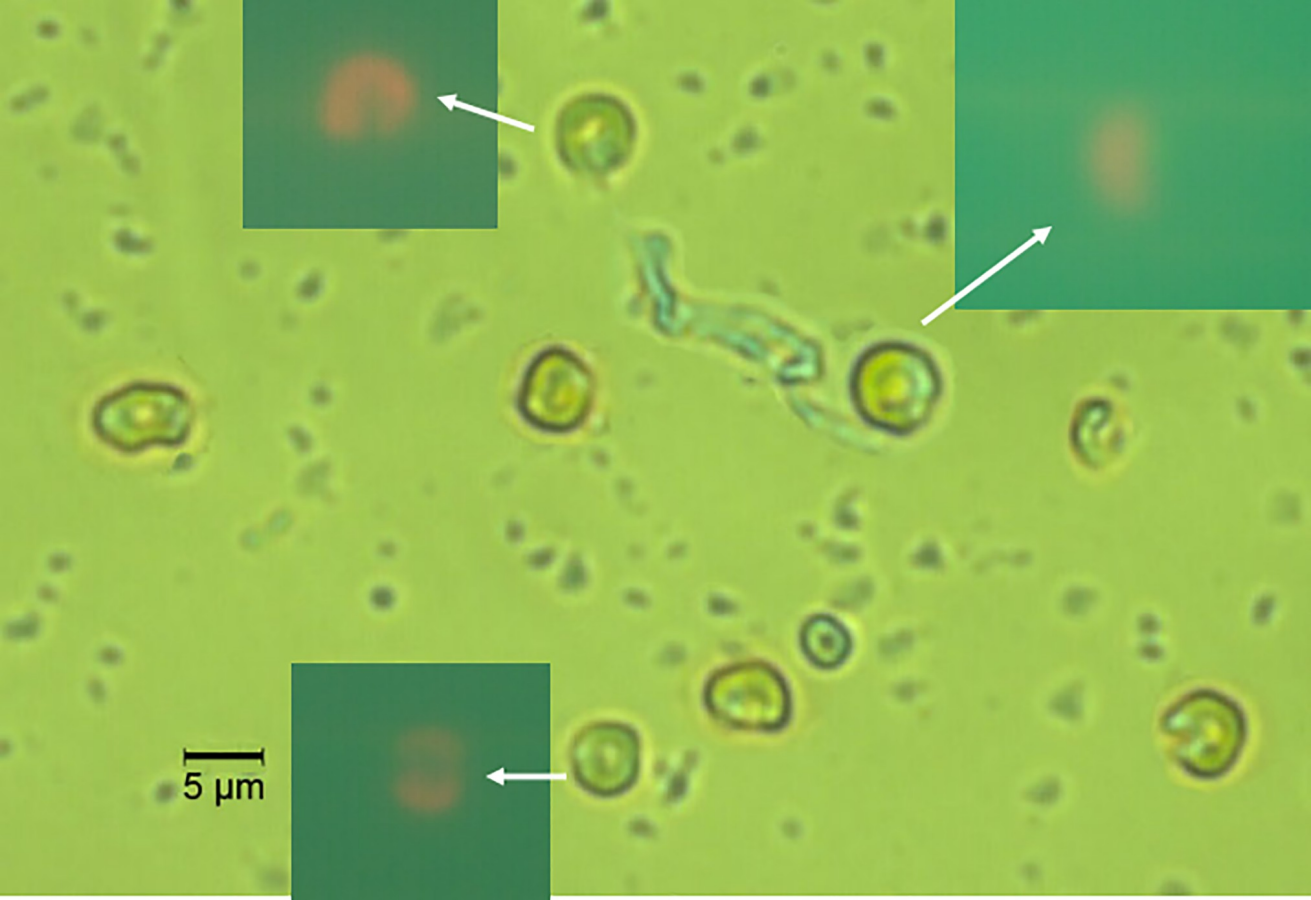
Micrograph of bacteriochlorophyll-containing bacterial cells detected from the 1000m at station E403. Insets: fluorescence (>515nm) of cells under excitation at 450-490nm. Thick arrow indicates the broken points in the cells.

The cells sorted by the flow cytometer from station E403 at 1000 m depth appeared to be rod-shaped but curved up to nearly a circle of approximately 2–5 μm in diameter and about 1μm in thickness (Fig 3). Red fluorescence of the cells detected by flow cytometer was also observed under the epifluorescence microscope (inset of Fig 3).

### Molecular analysis

**Fig 4:**
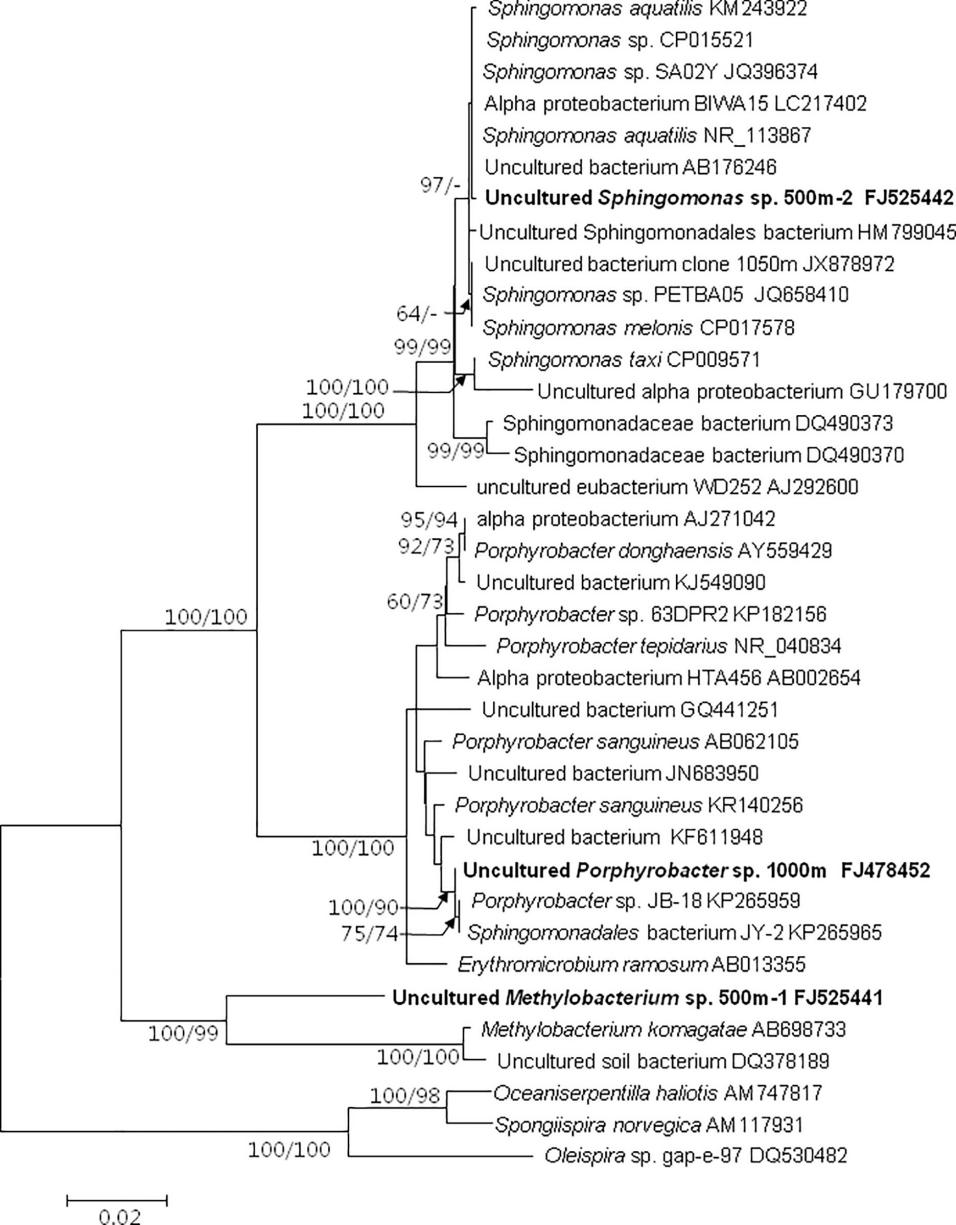
Phylogram of detected bacteria and related lineages based on 16S rDNA. **Sequence obtained in this study is bold-typed.** Support of nodes is based on bootstrap values of NJ/ML with 1000 and 500 resamplings, respectively. Only values greater than 60% are shown. If only one of the two phylogenetic methods yielded significant support, the other is shown with ‘‘-”. *Oleispira* sp. was used as an outgroup to root the tree.

PCR using the 16S rDNA primers yielded the expected 1.5 kb-long amplicons from both the 1000 m and 500 m samples, a size expected for bacterial 16S rDNA. Parallel PCR reactions without sample addition yielded no products, showing that possible contamination during the pipetting of PCR ingredients did not occur. After cloning and sequencing the PCR products, BLAST analysis was conducted and showed that the 1000 m sample contained a bacterium (clone Pac1000QI-1; GenBank Accession number FJ478452) with 99% sequence identity to various strains of *Porphyrobacter sanguineum* (formerly *Agrobacterium sanguineum*; GenBank Accession number AB062105) [15], including marine strains (GenBank Accession number KP265965, KP265959). Phylogenetic analysis also revealed affiliation of this 16S rDNA clone with *Porphyrobacter sanguineum* (Fig 4) among various bacterial species included in the analysis. The BLAST analysis also showed that the sequences retrieved from the 500 m sample contained a clone that was 99% identical to *Sphingomonas aquatilis* (GenBank Accession number KM243922, NR_113867), *Sphingomonas melonis* (GenBank Accession number CP017578), and *Sphingomonas* sp., all of which were isolated from petroleum contaminated environments (GenBank Accession number JQ658410, JQ396374, JX878972) [27,28]. The sequences also contained a clone that was related to *Methylobacterium komagata* (GenBank Accession number AB698733) [29], a bacterium isolation from oil polluted soil (GenBank Accession number DQ378189). *Porphyrobacterium*, *Sphingomonas*, and *Methylobacterium* all belong to the bacteriochlorophyll-containing subgroup of *Alphaproteobacteria*. The phylogenetic tree of 16S rDNA included 34 sequences from Genbank, in addition to the sequences obtained in this study. The topologies of the tree inferred from the dataset using Neighbor Joining (NJ) and Maximum Likelihood (ML) indicated clear and well-supported separation of three groups (Fig 4). The phylogeny placed *Sphingomonas* as a distinct lineage, well separated from *Porphyrobacterium* (GenBank Accession number FJ478452, KP265959, AB062105, KR140256), and *Methylobacterium* species (GenBank Accession number FJ525441, AB698733) (Fig 4).

## Discussion

There are many reports on infra-red fluorescence of AAP bacteria or bacteriochlorophyll under the microscope [25, 26], but no records from a flow cytometer are available due to technical challenges in detecting infra-red fluorescence that is characteristic of bacteriochlorophyll. However, the FACSAria flow cytometer increasingly available is able to detect dim infra-red fluorescence (780/60-nm). In this study, we were able to observe the red/infra-red fluorescence on deep-sea samples by FACSAria flow cytometer (Fig 2B, 2C, 2E and 2F).

AAP bacteria are less recognized in the deep sea, the generally dark provinces of the ocean, than in surface waters. This is probably because they are not so common and sampling in deep waters has been relatively sparse on both temporal and spatial scales, easily missing these bacteria if their distribution is patchy and dynamic. Furthermore, as the abundance of bacteriochlorophyll-containing bacteria in deep water is generally low, these bacteria are difficult to detect directly using such common methods as fluorescent staining or molecular analysis without prior concentrating or sorting by a cell sorter. Additionally, bacteriochlorophyll-containing bacteria may escape detection by culturing techniques because most deep-water bacteria defy cultivation under regular bacterial culturing conditions. Although high-throughput 16S rDNA sequencing can reveal the high diversity of microbial diversity in the deep sea (e.g. Huber et al. 2007), few studies have targeted pigment-containing bacteria, thus limiting our knowledge of their diversity in the dark deep sea. Our result reported here demonstrates that flow cytometric cell sorting coupled with molecular analysis is a very useful approach to detect pigmented bacteria in the deep sea. Bacteriochlorophyll-containing bacteria were detected at two of the seven investigated deep sea stations in the South China Sea. 16S rDNA analysis revealed that the three different lineages of pigmented bacteria at these stations were closely related to *Porphyrobacter sanguineum*, *Sphingomonas* sp., and *Methylobacterium* sp., which belong to different subclasses of Alphaproteobacteria, with the first two in the α-4 subgroup and the last in the α-2 subgroup. Recent surveys also have identified *Sphingomonas* or *Methylobacterium* in 6000 m deep ocean environments (GenBank Accession number HM799045) [30], deep-sea hydrothermal areas at the Suiyo Seamount (GenBank Accession number AB176246) [31], as well as the glacial and subglacial environments in Antarctica (glacial ice, Lake Vostok accretion ice, cryoconite holes), Greenland (glacial and subglacial “silty” ice), China (glacial ice), and New Zealand (subglacial sediment) [32]. These pigmented bacteria likely possess various strategies to survive in hydrothermal vents, petroleum contaminated water or soil, or freezing cold deep sea and ice environments [27,28,31,32].

Members of the genus *Sphingomonas* are able to degrade a wide range of natural and xenobiotic compounds, such as polycyclic aromatic hydrocarbons [33–35]. Station E703, where *Sphingomonas* and *Methylobacte*rium were detected from the 500 m depth, is near the gas field, Baiyun Sag, located in the Pearl River Mouth Basin [36]. The hydrological measurements in our cruise showed that there was an upward vertical current at station E703 (SCSIO Open-Cruises physical oceanography data report in September, 2005), raising a possibility that the cell population was brought up with gas contaminated seawater (for the 500 m sample) from the nearby gas field.

However, it is unclear how *Porphyrobacter* sp. acquires energy in deep water beyond light’s penetration (euphotic zone). There are few reports of light availability below 500 m. It raises the question whether these bacteria were brought down from the eutrophic zone and only remain in the great depth temporarily. However, the fact that no cyanobacteria, which can also emit red and infra-red fluorescence, were detected from the sorted cell population by the universal 16S rDNA primers suggests that surface origination of the detected pigmented bacteria is unlikely. Furthermore, most of *Porphyrobacter* sp. cells appeared broken under the microscope (Fig 3), suggesting that the population was adapted to the deep-sea environment and could not tolerate the huge pressure change when it was brought up to the surface during sampling. The maximum inflow of Kuroshio into Bashi Channel and South China Sea occurs in September–October where a large portion of the channel is deeper than 1000 m [37]. Thus, the bacteriochlorophyll-containing bacteria detected at station E403 at 1000 m depth might have originated from the Bashi Channel hydrothermal vent area, which could be carried to station E403 by the Kuroshio inflow. This would be consistent with the finding of AAP bacteria from the black smoker plume water of Juan de Fuca ridge in the Pacific Ocean [2].

## Conclusion

This work investigated AAP bacteria distribution in deep sea. It revealed existence of bacteriochlorophyll-containing bacteria below 500 m from two of the stations surveyed in the South China Sea. The bacteria sorted from the 1000 m depth at station E403 belonged to the genus *Porphyrobacter* and those sorted from station E703 at 500 m depth were *Sphingomonas* and a *Methylobacterium-*like taxa. Future research using integrated approaches, such as flow cytometry and “Omics” methods in combination, is required in order to continue to improve our understanding of the physiologies of deep-sea AAP bacteria.
